# Appreciating the Good Things in Life During the Covid-19 Pandemic: A Randomized Controlled Trial and Evaluation of a Gratitude App

**DOI:** 10.1007/s10902-022-00586-3

**Published:** 2022-10-06

**Authors:** Noortje Kloos, Judith Austin, Jan-Willem van ‘t Klooster, Constance Drossaert, Ernst Bohlmeijer

**Affiliations:** 1 Department of Psychology, Health and Technology, Centre for eHealth and Wellbeing Research, University of Twente, P.O. Box 217, 7500 AE Enschede, The Netherlands; 2 School of Nursing and Midwifery, La Trobe University, Melbourne, Australia; 3 Behavioural, Management and Social Sciences Lab, University of Twente, Enschede, The Netherlands

**Keywords:** Gratitude, Well-being, Intervention, Mobile application, Positive psychology, Randomized controlled trial

## Abstract

The Covid-19 pandemic has had many negative consequences on the general public mental health. The aim of this study was to test the effectiveness of and satisfaction with an app with gratitude exercises to improve the mental health of people with reduced mental well-being due to the Covid-19 pandemic, as well as potential mechanisms of well-being change and dose–response relationships. A two-armed randomized controlled trial design was used, with two groups receiving the 6-week gratitude intervention app either immediately (intervention group, *n* = 424) or after 6 weeks (waiting list control group, *n* = 425). Assessments took place online at baseline (T0), six weeks later (T1) and at 12 weeks (T2), measuring outcomes (i.e., mental well-being, anxiety, depression, stress), and potential explanatory variables (i.e., gratitude, positive reframing, rumination). Linear mixed models analyses showed that when controlled for baseline measures, the intervention group scored better on all outcome measures compared to the control group at T1 (*d* = .24–.49). These effects were maintained at T2. The control group scored equally well on all outcome measures at T2 after following the intervention. Effects of the intervention on well-being were partially explained by gratitude, positive reframing, and rumination, and finishing a greater number of modules was weakly related to better outcomes. The intervention was generally appealing, with some room for improvement. The results suggest that a mobile gratitude intervention app is a satisfactory and effective way to improve the mental health of the general population during the difficult times of a pandemic.

## Introduction

Recent literature has paid much attention to the multitude of negative consequences of the Covid-19 pandemic, extending beyond getting ill. The constant threat of becoming infected combined with government-imposed restrictions has had major impact on the mental health of the general public, evident in the reduced mental well-being, and increased anxiety, depression and stress levels that have been reported (e.g., Agha, 2021; Paredes et al., 2021). Even when life will gradually get back to normal, a certain extent of restrictions will likely remain due to breakthrough infections. Easy to use large-scale low-intensity interventions for the general population are needed to improve mental health, also beyond the pandemic (Huppert, 2004; Schotanus-Dijkstra et al., 2019). Positive psychological interventions, such as gratitude exercises, could help to reduce distress, maintain mental well-being, and develop psychological resources needed to cope with the psychosocial consequences of the pandemic (Serlachius et al., 2021; Waters et al., 2021). The current study tested whether a gratitude app provides an effective and satisfactory intervention to improve the mental health of people with reduced well-being during the Covid-19 pandemic.

Sustainable mental health can be seen as the combination of low ill-being and high well-being and the presence of the ability to adapt (Bohlmeijer & Westerhof, 2021). Indicators of mental ill-being are for example chronic stress and distress. Mental well-being, on the other hand, is a combination of *feeling well*: positive emotions and life satisfaction, and *doing well*: positive functioning of the individual and in society (Keyes, 2002). Mental well-being and ill-being are related but differentiated continua: a person without ill-being does not necessarily experience high well-being (Lamers et al., 2011a; Westerhof & Keyes, 2010). However, well-being reduces the risk of incidence of mental ill-being (e.g., Schotanus-Dijkstra et al., 2017) and sustainable positive mental health has shown to have a buffering effect on ill-being, a bolstering effect on continued mental health, as well as a building effect on growth during the pandemic (Waters et al., 2021).

Gratitude may be an important resource to improve and protect mental health. Gratitude refers to the awareness of the positive aspects of life and the goodness of others (Sansone & Sansone, 2010). It can be seen as a positive emotion that one experiences when receiving benefit from someone (McCullough et al., 2002), as a mood that lingers on for a longer period of time (McCullough et al., 2004), and eventually as a general dispositional tendency (Watkins et al., 2003; Wood et al., 2009). Gratitude has been linked to both improved well-being and reduced ill-being (Wood & Joseph, 2010). Various underlying mechanisms have been proposed to explain the effect of gratitude on mental health, such as through improving positive emotions (Lambert et al., 2012), positive relationships (Algoe & Zhaoyang, 2016), and effective coping (Wood et al., 2007). Gratitude has shown to be positively related to coping strategies such as seeking social support, planning, and positive reframing (Lambert et al., 2012) and negatively to rumination (Heckendorf et al., 2019). Studies suggest that gratitude has potential to promote resources and reduce barriers for successful adaptation to difficult life-events and life-circumstances such as a pandemic (Bernabe-Valero et al., 2021; Bohlmeijer & Westerhof, 2021; Jans-Beken, 2021; Mead et al., 2021).

Various exercises, such as gratitude journaling and writing gratitude letters, have been developed to promote gratitude (Emmons & McCullough, 2003; Emmons & Stern, 2013). Meta-analyses have shown that these gratitude interventions are effective in improving mental health, although effects are generally small to moderate, and inconsistent (Davis et al., 2016; Dickens, 2017). Several modifications have been proposed to improve the effectiveness, such as increasing the duration of the intervention, and including multiple exercises, and it has been suggested to examine effects in people who are experiencing various levels of distress (Bohlmeijer et al., 2022) . Furthermore, gratitude interventions could benefit from delivery in a mobile digital format (mHealth). Online and mobile app formats can have several advantages, such as availability, accessibility, flexibility, adaptability, easy scalability, cost-effectiveness, integration in daily life, and the opportunity to include persuasive elements to improve motivation and adherence (e.g., Bidargaddi et al., 2018; Mohr et al., 2010; Olff, 2015; Wei et al., 2020). These advantages make an app-based gratitude intervention especially attractive for implementation in the general public. A handful of studies have investigated the effectiveness of such gratitude apps on body satisfaction in women (Fuller-Tyszkiewicz et al., 2019), and on mental health in younger children (Lau et al., 2020) and high school students (Bono et al., 2020). One study showed that a gratitude app combined with an online gratitude training and adherence-focused guidance (human support) decreased anxiety and depression in adults with elevated levels of repetitive negative thinking, which was mediated through reduced rumination (Heckendorf et al., 2019). Yet, more research is clearly needed on the effectiveness of self-help gratitude interventions aimed at the general public, without barriers that limit their reach such as additional guidance.

The aim of the current study was to test the effectiveness of and satisfaction with an app with gratitude exercises to improve mental health of people with reduced well-being due to the Covid-19 pandemic. The app was based on an extensive 6-week gratitude intervention that has previously been shown effective in improving mental well-being and gratitude in people with low to moderate well-being and mild distress (Bohlmeijer et al., 2020). Besides effectiveness, the current study investigates gratitude and coping as possible mechanisms of change and examines dose–response relationships and the appeal of the intervention for the general population.

## Methods

### Design

This study used a two-armed randomized waiting list controlled design to test the effectiveness of a 6-week gratitude intervention app. Assessments for all participants took place online at baseline (T0), 6 weeks later (T1), and at 12 weeks (T2). The intervention group received the gratitude intervention app following T0, and the wait list control group received the app following T1 (effectively no longer serving as true control at T2). This design was chosen as we did not consider it ethically justified to offer people who are seeking support to improve their well-being in the context of a pandemic crisis a sham or neutral intervention, and we wanted to give all participants access to the intervention as soon as possible. Participants were not blinded for allocation. This study was approved by the Ethics Committee of the University of Twente (no. 201071) and was registered at the Netherlands Trial Register (trial NL8856).

### Gratitude Intervention App

**Fig. 1:**
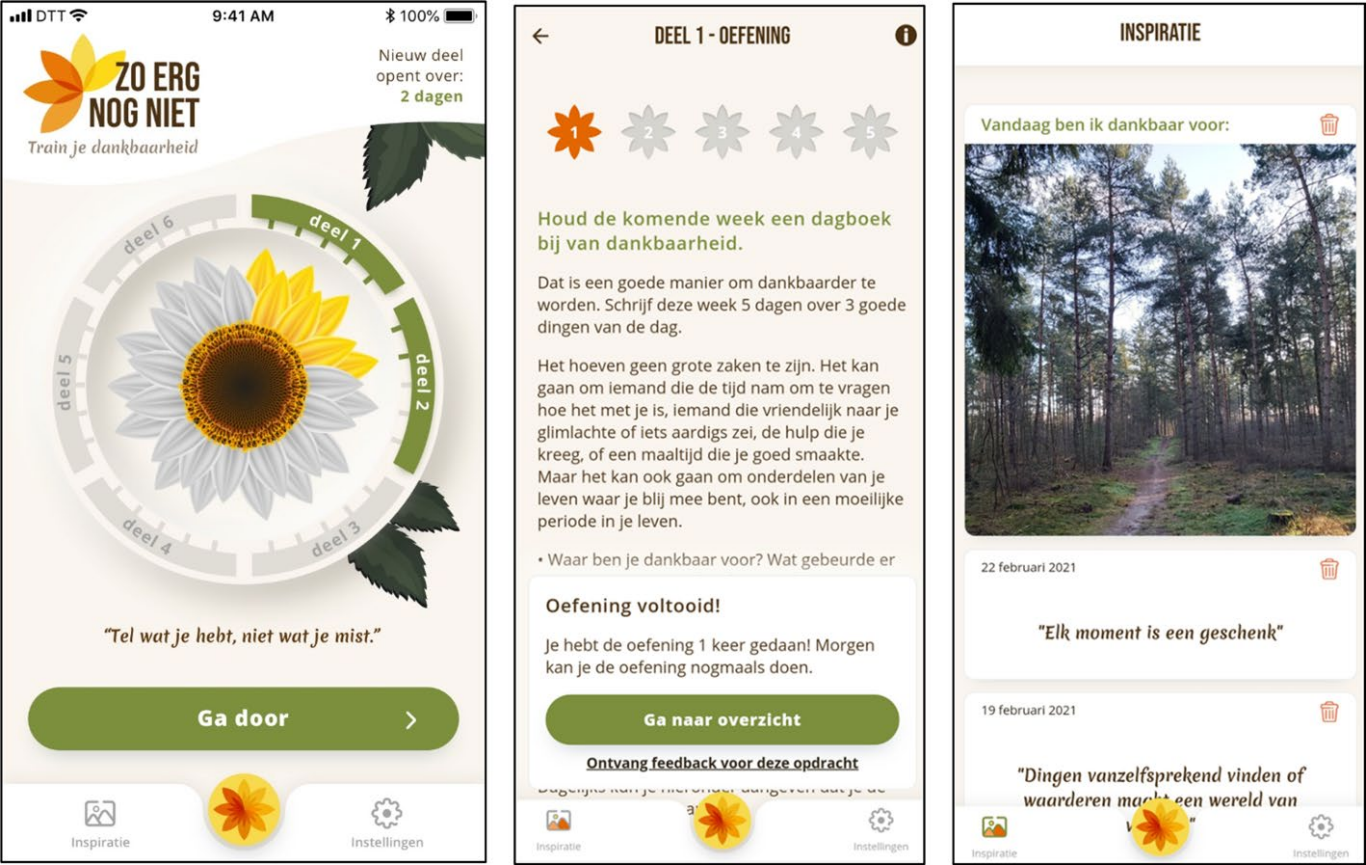
Screenshots ZENN gratitude app: home screen, exercise, photo functionality

The gratitude intervention app “ZENN” (Dutch: *“Zo Erg Nog Niet”,* translated *“Not That Bad”)* is a progressive web app (PWA), that users can access on their smartphone, tablet or computer with a login code (Fig. 1). The app was specifically designed for this study. The content of the gratitude app was based on the e-mail-delivered intervention that has previously been shown effective (Bohlmeijer et al., 2020), and the design, navigation, structure, and persuasive elements of the app were based on a previous, user-based designed self-compassion app (Austin et al., 2022). Six modules covered psycho-education on several aspects of gratitude in both short movie clips and in text: (1) Seeing and appreciating the positive, (2) Appreciating daily things, (3) Expressing gratitude, (4) Discovering positive consequences of adversity, (5) Awareness of impermanence, (6) Gratitude as life attitude. Each module had one evidence-based gratitude writing exercise suitable for daily repetition (see Bohlmeijer et al., 2020). Users were advised to spend about 10–15 min a day on writing, 5 days a week (50–75 min per week). The app included several persuasive elements that can be used in web-based interventions (Kelders et al., 2012). The homepage of the app visualised a sunflower, which was gradually colourized upon progressing through the training (self-monitoring and liking). The app included daily inspirational quotes and reminders to do the exercise and the opportunity to upload one photo a day of something one is grateful for. Users received a reward upon completing an exercise in the form of a coloured flower, and received suggestions in the form of tailored automated feedback after completing an exercise twice. The app was completed in chronological order (tunnelling), with a new module accessible upon completing a weekly exercise five times per week (rehearsal). Email-based technical support was provided for installing or log-in issues.

### Procedure

The study took place between January and May 2021, a time when there were no or only limited vaccines available, and lockdown, curfew, working from home, (partly) home-schooling, travel restrictions and social restrictions were implemented in the Netherlands and Belgium. Participants were recruited in the general Dutch and Belgian population via various social media of the University (LinkedIn, Instagram, Facebook), via articles in regional and national newspaper, and radio-interviews. The recruitment message was: “*Are you experiencing less well-being due to the corona crisis? Could you use some positivity and resilience?*”. Interested people were informed about the study and could sign up via a study website. After providing informed consent, an online screening questionnaire could be completed to determine eligibility. Participants who were not eligible to participate due to exceeding anxiety and/or depressive scores (see eligibility criteria below) were informed by email to contact their physician if needed. This criterium was adopted to prevent potential participants misinterpreting the intervention as an effective alternative to seeking treatment from a mental health professional to treat their anxiety- or depressive symptoms. Eligible participants were invited to complete the baseline questionnaire online, after which they were randomly assigned to either the intervention group or the waiting list control group, using randomizer.org.

### Sample

Participants were Dutch and Flemish adults in the general population who felt they suffered from reduced well-being due to the corona crisis. A power analysis showed that when taking into account a 30% loss to follow-up, 224 people divided over the two groups were needed to have a power of 0.80 to obtain an effect size of at least *d* = 0.45 on well-being (Bohlmeijer et al., 2020; Bolier et al., 2013). However, given the exceptional situation of the COVID-19 pandemic in which we felt it our ethical responsibility to give people access to the intervention as soon as possible, we decided to open study participation to all eligible people over the course of a 2-week register period.

Inclusion criteria were: age of 18 years or older; sufficient command of the Dutch language; in possession of an email address and smartphone or tablet with sufficient internet connection; willing to do a 15-min gratitude exercise daily for 6 weeks. Exclusion criteria included the presence of severe anxiety symptoms (i.e. a score of ≥ 15 on the Generalized Anxiety Disorder-7; Spitzer et al., 2006) and moderately severe or severe depressive symptoms (i.e. ≥ 15 on the Patient Health Questionnaire; Kroenke et al., 2001).

**Fig. 2:**
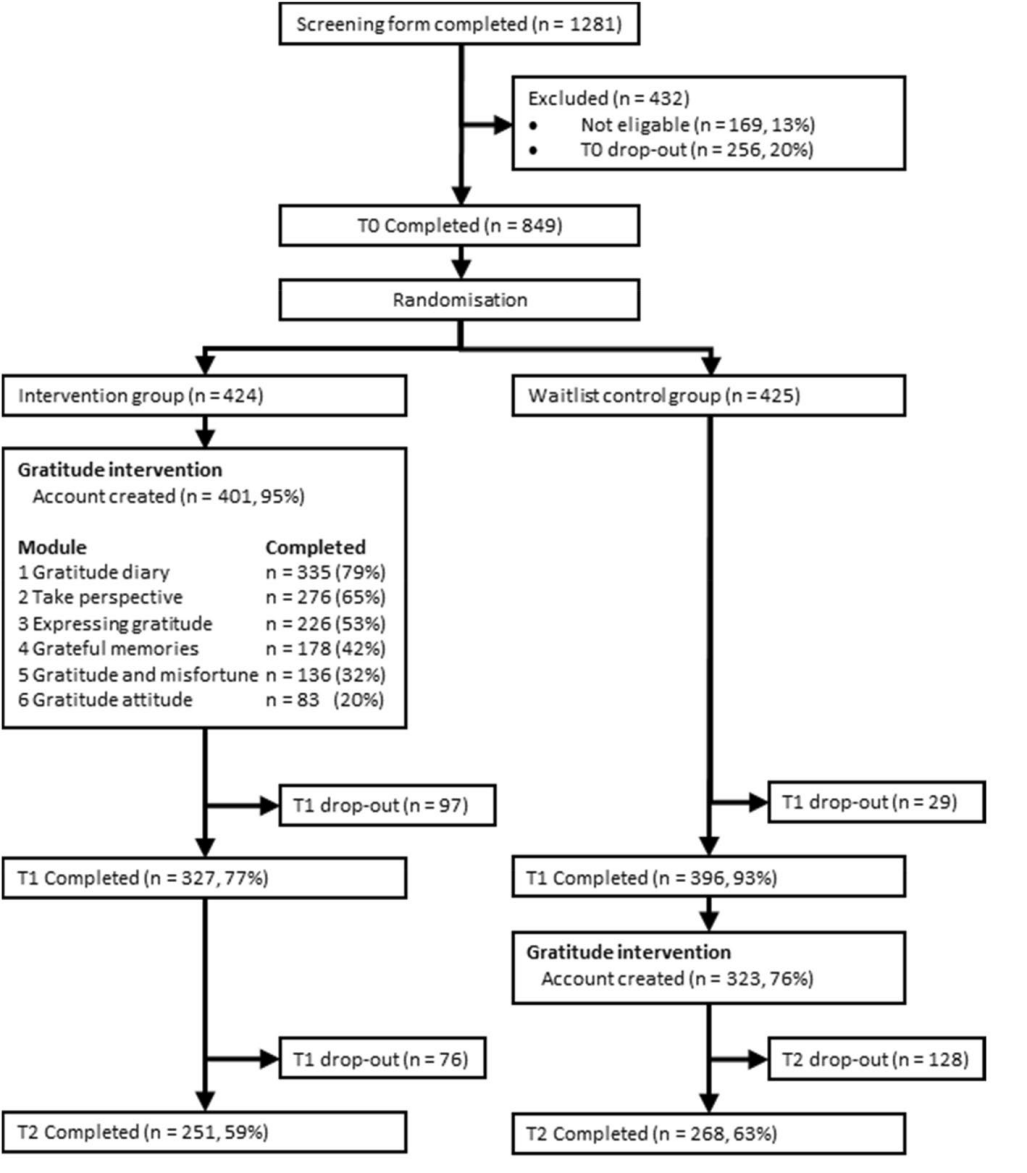
Flowchart of participants

Participants were recruited via radio items (38%), articles in (online) papers or journals (34%), or via recommendations by others (11%). Figure 2 shows that a total of 1281 filled out the screening form. However, 175 participants were not eligible to participate because of anxiety or depressive symptoms above the cut-off (*n* = 169, 13%), or because they were not willing to do the 15-min exercises daily (*n* = 4). An additional of 256 participants (20%) dropped out between screening and T0 (reasons unknown). The final sample consisted of 849 participants at baseline (intervention group *n* = 424, waiting list control group *n* = 425).

### Measurements

Demographic data and the experienced impact of Covid-19 (e.g., corona history, influence on well-being) were gathered at baseline. Questionnaires measuring outcomes (well-being, anxiety, depressive feelings, stress) and possible explanatory variables (gratitude, positive reframing, and rumination) were administered online at each assessment (T0, T1, and T2). Intervention participants provided self-reported adherence, and app evaluations (i.e., satisfaction and engagement) following the intervention (T1). Gratitude app usage (i.e., registration date, frequency of accessing the app, photo uploading frequency, last module finished, date of last visit) were logged.

#### Well-being

Well-being was measured with the Mental Health Continuum Short Form (MHC-SF; Keyes, 2002; Lamers et al., 2011a), consisting of 14 items. The scale measures *‘In the past month, how often did you…’* experience emotional well-being (3 items, e.g., *‘…feel happy?’*), social well-being (5 items, e.g., *‘…people are basically good’*), and psychological well-being (6 items, e.g., *‘…feel confident to think or express your own ideas and opinions?’*). Items are scored on a scale from 0 *never* to 5 *every day*, and an average total scale is computed, with high scores indicating high levels of well-being. The scale has shown good psychometric properties (Lamers et al., 2011a). In the current sample, the internal reliability was good, with α = 0.89 (subscale reliability ranged from α = 0.71–0.82).

#### Anxiety

Anxiety symptoms during the past two weeks was measured with the 7-item Generalized Anxiety Disorder (GAD-7; Spitzer et al., 2006). An example item is ‘*Worrying too much about different things'*. Items were scored on a scale from 0 *not at all* to 3 *nearly every day*. A total sum score is calculated, ranging from 0–21, with higher scores indicating more symptoms of generalized anxiety. Severity score cut-offs are provided, with 0–4 indicating minimal, 5–9 mild, 10–14 moderate, and 15–21 severe anxiety. The Dutch version of this scale has previously shown good psychometric properties (Donker et al., 2011) and the current sample showed acceptable to good reliability (T0 α = 0.77, T1 α = 0.87).

#### Depression

Depressive symptoms during the past two weeks was measured with the 9-item Patient Health Questionnaire (PHQ-9; Kroenke et al., 2001), with each item related to one criterium for depression (e.g., ‘*Little interest or pleasure in doing things’*). Items were scored on a scale from 0 *not at all* to 3 *nearly every day*. A total sum score is calculated, with 0–4 indicating minimal, 5–9 mild, 10–14 moderate, 15–19 moderately severe, and 20–27 severe depressive symptoms (Kroenke et al., 2001). The current sample showed acceptable to good reliability (T0 α = 0.72; T1 α = 0.82).

#### Stress

The 10-item Perceived Stress Scale (PSS; Cohen et al., 1983) was used to measure the degree to which participants appraised situations in life as stressful in the past month. An example question is: ‘*In the last month, how often have you felt nervous or stressed?’*. Items are scored on a scale from 0 *never* to 4 *very often*, which are summed to one total score ranging from 0 to 40, with high scores indicating more perceived stress. This scale has shown acceptable psychometric properties (Lee, 2012), with good reliability of α = 0.85 in the current sample.

#### Gratitude as Trait

The six-item Gratitude Questionnaire (GQ-6; McCullough et al., 2002) was used to measure gratitude as a stable trait. An example question is ‘*I have so much in life to be thankful for’*, with all items scored on a scale from 1 *strongly disagree* to 7 *strongly agree*. A total mean score is computed, with high scores indicating higher levels of grateful disposition. The Dutch version of the scale has shown good psychometric properties (Jans-Beken et al., 2015), and the current sample showed acceptable reliability, with α = 0.74.

#### Gratitude as Mood

A four-item measure of grateful mood (McCullough et al., 2004) was used, measuring the extent to which participants felt grateful, were aware that life was good for them, appreciated simple things in life, and were grateful for what others mean in their lives, during the past 24 h. Items are scored on a scale from 1 *strongly disagree* to 7 *strongly agree,* with higher total mean score indicating higher level of grateful mood. The Dutch version of these questions have shown good reliability (Bohlmeijer et al., 2020), and in the current sample a good reliability of α = 0.87 was found.

#### Positive Reframing

The 4-item Positive Reinterpretation and Growth subscale of the Coping Orientations and Problems Experienced inventory (COPE; Carver et al., 1989) was used to measure positive reframing. An example item is *‘I've been looking for something good in what is happening’,* with items scored on a scale from 1 *I haven’t been doing this at all* to 4 *I’ve been doing this a lot*. A total sum score is computed, with higher scores indicating more positive reframing. This scale has shown good psychometric properties (Monzani et al., 2015). The current sample showed good reliability, with α = 0.85.

#### Rumination

Rumination was measured with the 15-item Perseverative Thinking Questionnaire (PTQ; Ehring et al., 2011), asking participants to indicate to what extend they engage in repetitive negative thoughts when recalling experiences and problems (e.g., ‘*Thoughts come to my mind without me wanting them to*’). Items are scored on a scale from 0 *never* to 4 *almost always*, and a total sum score is computed ranging from 0 to 60, with high scores indicating a high degree of rumination. The Dutch version of the scale has shown good psychometric properties (Ehring et al., 2012) and the current sample showed excellent reliability with α = 0.95.

#### Self-reported App Adherence

Self-reported adherence was measured, asking *how many days per week* participants used the app on average, and *how much time per day* they spend on the exercises on average.

#### App Evaluations

Overall satisfaction with the training was measured with the 8-item client satisfaction questionnaire (CSQ; Attkisson & Zwick, 1982). Items are scored on a scale from 1 to 4, with various labels fitting each item (e.g., *how would you rate the quality of [the app]?* 1 *poor to* 4 *excellent)*. Total sum scores are calculated, ranging from 8 to 32, with higher scores indicating greater satisfaction with the app. The scale had excellent reliability, with α = 0.94 in the intervention group in the current sample.

Intervention participants were asked to indicate which module(s) of the app they felt contributed to experiencing more positivity. Finally, satisfaction with specific aspects of the app were measured with 10 items. Five items asked for satisfaction with language, design, usability, amount of text, and number of modules on a scale from 1 *bad* to 5 *very good*. Further, five items asked for the extent to which the texts, video’s, exercises, daily quotes and notifications were appealing, on a scale from 1 *not* to 4 *completely*.

### Analyses

Analyses were conducted using SPSS 25.0, with the alpha level set to 0.05. Descriptive statistics of socio-demographic variables and experienced impact of the corona-crisis were compared between intervention and control group, using two-tailed independent sample t-tests and χ^2^-tests.

#### Drop-Out and Adherence

To determine whether intervention and control groups differed in frequency of T1 drop-out (completing only baseline questionnaires), or T2 drop-out (completing only T0 and T1 questionnaires) two χ^2^-tests were conducted. We compared T1 questionnaire drop-outs to T1 completers on demographics, experienced impact of the corona crisis, and baseline outcome measures, using independent sample t-tests and χ^2^-tests.

For intervention group participants, log data were missing for 6 participants (1%) who deleted their accounts. Self-reported adherence in terms of time spent on the app were described. Adherence was set to finishing at least four modules, in line with previous research showing gratitude mediates intervention effects on mental well-being from 4 modules onward (Bohlmeijer et al., 2022) and spending on average at least 10 min per day for at least 5 days a week on exercises, as was communicated with users in the app.

#### Effectiveness

To test the effectiveness of the intervention, we first conducted paired-sample t-tests to examine whether the intervention group improved on outcome and explanatory variables. Furthermore, modified intention-to-treat analyses were conducted with the Linear Mixed Models (LMM) procedure, including all participants who completed the baseline questionnaire. The LMM analyses included the fixed effect of condition (intervention vs. control), T1 measures as dependent variable, and T0 measures as control variable for each outcome measure (i.e., well-being, anxiety, depressive symptoms, stress) and explanatory variables (gratitude, positive reframing, and rumination), with restricted maximum likelihood as the estimation method. The effect size of Cohen’s *d* was calculated by dividing the T1 mean difference of the estimate marginal means of the intervention and control condition by the pooled standard deviations, with Cohen’s *d* < 0.33 as small, 0.33–0.55 as moderate and > 0.55 as large effects (Lipsey & Wilson, 1993).

Since the control group received the intervention between T1 and T2, we checked whether scores of the control group improved to the level of the intervention group, and whether the effects in the intervention group remained at T2. For this, we conducted LMM analyses on T2 between-group differences with T0 measures as control. Further, longer-term efficacy was examined by employing LMM within-subject comparisons with the intervention group only, including the fixed effect of time as repeated measure (T1 vs. T2), for each outcome measure. Completers-only analyses showed similar results and are therefore not reported.

To further explore whether the intervention was effective for people with various levels of distress (anxiety and depression), we conducted two sensitivity analysis using the PROCESS tool (version 4) (Hayes, 2012). Baseline anxiety and depression scores were each divided in three categories based on questionnaire guidelines, representing minimal, mild, and moderate anxiety and depression (Kroenke et al., 2001; Spitzer et al., 2006). A simple moderation test was conducted with condition as X (1 for intervention, 0 for control group), T0-T1 change scores in well-being as Y, and the categorized baseline measure of anxiety or depression as W (moderator). A significant highest order unconditional interaction effect would lead to inspection of the conditional effects.

#### Path Analyses

The explanatory role of gratitude and coping (positive reframing, and rumination) in the intervention effect on well-being were assessed with the PROCESS tool. Simple path analyses were conducted with condition as X, T0-T1 change scores in well-being as Y, and T0-T1 change scores of one of the possible explanatory variables as M. Unstandardized regression coefficients were calculated for each path in the path model: path a (effect of X on M), path b (effect of M on Y, controlled for X), path c (total effect of X on Y), and path c’ (direct effect of X on Y controlled for the effect of M). The indirect effect of X on Y through M is calculated as the product of a and b (ab) of which the bias-corrected (BC) 95% CI’s were based on 10,000 bootstrapped resamples (Hayes, 2012). Explanatory path is inferred when the 95% CI does not include zero.

#### Dose–Response Relations

To assess possible dose–response relationships in the intervention group, Pearson correlations were calculated between the number of modules finished and T0-T1 change-scores in well-being, anxiety, depressive feelings, and stress. Correlations of* r *≤ 0.29 indicated weak, *r* ≤ 0.49 moderate and *r *≤ 0.50 strong relations (Cohen, 1988). To provide further insight in the nature of these relationships, we included a visual representation of the number of modules finished and associated effect sizes of the T0-T1 differences (Cohen’s *d* based on the LMM estimated marginal means for each group).

#### App Evaluations

Intervention group participants’ evaluation of the app were analysed using descriptive statistics.

## Results

### Participants

Table 1 shows the baseline characteristics of participants. Participants had a mean age of 53 years (*SD* = 15, range 18–83 years). Most participants were female (80%), Dutch (78%) or Belgian (20%), highly educated (81%), employed (65%), married or in civil partnership (57%), living with a partner (41%) or with partner and children (27%). At baseline, about 21% of participants felt they belonged to a Covid-19 risk group, and a small group had been infected with the corona-virus (7%), had a loved-one admitted to the hospital (3%), or lost a loved one to infection with Covid-19 (6%). Most participants described that the Corona pandemic had a moderate influence on their well-being. Intervention and control group did not differ on any of the demographic variables, other than that a higher percentage of participants in the intervention group where Dutch compared to the control group.

**Table 1:** Baseline characteristics of participants in the control group and intervention group and total sample

	Intervention (*n* = 424)	Control (*n* = 425)	Total (*n* = 849)
Age, *M* (*SD*)	53.2 (14.7)	52.5 (14.3)	52.9 (14.5)
Gender, *n *(%)			
Female	332 (78)	345 (81)	677 (80)
Male	92 (22)	77 (18)	169 (20)
Not defined	-	3 (1)	3 (0)
Nationality *n* (%)			
Dutch	350 (83)	316 (74)	666 (78)
Belgian	66 (16)	107 (25)	173 (20)
Other	8 (2)	2 (0)	10 (1)
Education, *n* (%)			
Low	48 (11)	59 (14)	107 (13)
Intermediate	30 (7)	26 (6)	56 (7)
High	346 (82)	340 (80)	686 (81)
Employment, *n* (%)			
On payroll or entrepreneur	275 (65)	276 (65)	551 (65)
Retired	96 (23)	85 (20)	181 (21)
Unemployed, volunteering	39 (9)	52 (12)	91 (11)
Student	14 (3)	12 (3)	26 (3)
Marital status, *n* (%)			
Married or civil partnership	228 (54)	255 (60)	483 (57)
Never been married	108 (26)	97 (23)	205 (24)
Divorced or widowed	88 (21)	73 (17)	161 (19)
Living situation, *n* (%)			
With partner or LAT	176 (42)	168 (40)	344 (41)
With partner and child(ren)	111 (26)	122 (29)	233 (27)
Alone	96 (23)	94 (22)	190 (22)
With child(ren)	17 (4)	21 (5)	38 (5)
With parent(s) or others	22 (5)	15 (4)	37 (4)
Corona risk group, *n* (%)			
No	331 (78)	324 (76)	655 (77)
Yes	88 (21)	93 (22)	181 (21)
Undefined	5 (1)	8 (2)	13 (2)
Corona history, *n* (%)			
No history	199 (47)	194 (46)	393 (46)
Tested	132 (31)	139 (33)	271 (32)
Isolation	65 (15)	62 (15)	127 (15)
(Probably) infected	28 (7)	30 (7)	58 (7)
Corona history loved ones, *n* (%)			
No history	100 (24)	95 (22)	195 (23)
Tested	112 (26)	101 (24)	213 (25)
Isolation	122 (29)	151 (36)	273 (32)
(Probably) infected	50 (12)	41 (10)	91 (11)
Admitted to hospital or IC	16 (4)	13 (3)	29 (3)
Deceased	24 (6)	24 (6)	48 (6)
Influence Corona crisis on well-being (1–5), *M* (*SD*)	2.7 (0.8)	2.8 (0.9)	2.8 (0.8)
Raw baseline mean scores, *M* (SD)			
Well-being	2.7 (0.8)	2.8 (0.8)	2.7 (0.8)
Anxiety	6.5 (3.2)	6.7 (3.2)	6.6 (3.2)
Depression	5.9 (3.2)	6.2 (3.5)	6.1 (3.3)
Stress	17.6 (5.5)	17.6 (5.4)	17.6 (5.4)
Explanatory variables			
Gratitude			
As trait	5.3 (0.9)	5.4 (0.9)	5.4 (0.9)
Mood	4.8 (1.2)	5.0 (1.2)	4.9 (1.2)
Positive reframing	11.6 (2.9)	12.0 (2.7)	11.8 (2.8)
Rumination	30.8 (9.9)	30.8 (9.9)	30.8 (9.9)

Table 1 shows that at baseline, the raw mean well-being scores of participants in both groups were slightly lower than the Dutch national norm-group (*M* = 2.98, Lamers et al., 2011b). Further, at baseline participants scored on average in the mild anxiety and mild depressive feelings range, with a group of *n *= 160 (19%) and *n* = 148 (17%) experiencing moderate symptoms of anxiety and depression, respectively (Kroenke et al., 2001; Spitzer et al., 2006). Groups differed at baseline on three baseline explanatory variables, with the intervention group scoring lower on gratitude (as stable trait and as mood), and positive reframing compared to the control group (respectively *t* (847) = 2.33, *p* = 0.02; *t* (847) = 2.36, *p* = 0.02; *t* (847) = 2.07, *p* = 0.04). Groups did not differ on any of the baseline outcome measures nor on the baseline explanatory variable of rumination.

### Drop-Out and Adherence

T1 drop-out (15%) was higher in the intervention group (*n* = 97, 11%) than in the control group (*n *= 29, 3%, χ^2^(1) = 42, *p* < 0.001), while T2 dropout (24%) was higher in the control group (*n* = 128, 30%), than in the intervention group (*n* = 76, 18%; χ^2^(1) = 8.1, *p* = 0.005). T1 drop-outs tended to have fewer app modules finished than completers. T1 drop-outs tended to be a bit younger (*M* = 48.7, *SD* = 15.5) than completers (*M* = 53.6, *SD* = 14, *t *(847) = 3.48, *p* = 0.001), and a relatively high percentage of T1 drop-outs lived with partner and children (39% of drop-outs, compared to 28% in the total group (χ^2^(4) = 17, *p* = 0.002). T1 drop-outs did not differ from T1 completers on any of the baseline outcome variables.

Log-data showed that most participants (*n* = 401, 95%) of the intervention group created an app account. Participants opened the app on average 49 times (*SD *= 44.7, range 0–297 times), exceeding the 30 times needed to finish all exercises, and uploaded on average 7 photographs (*SD *= 9.7, range 0–40). Most participants (75%) indicated using a paper notebook for the exercises, others used their smartphone (12%), the computer (8%), or tablet (5%), and a few did not write anything down (3%). About one third of participants arrived at the final module, and a large minority (42%) finished at least half of the intervention (Fig. 2). Most participants reported using the app for about 5 or more days per week (58%), and spending on average 10 min or more per day doing the exercise (70%). Overall, 122 participants (29%) adhered to the intended app usage (i.e., 4 or more modules finished, spending at least 10 min per day for at least 5 days per week on the intervention).

### Effectiveness

It was expected that the gratitude app would improve mental well-being. First, the control group improved in one outcome measure between T0 and T1 (well-being* t*(395) = 2.4, *p* = 0.02) and three explanatory variables (gratitude as trait: *t*(392) = 3.0, *p* = 0.003; gratitude as mood *t*(392) = 3.7, *p* < 0.001; rumination *t*(392) = 6.3, *p* < 0.001). The intervention group, however, improved more and with significant improvements on all outcome measures between T0 and T1 (well-being *t*(326) = 11.6, *p* < 0.001; anxiety *t*(325) = 3.2, *p* = 0.002; depression *t*(324) = 4.9, *p* < 0.001; stress *t*(325) = 9.15, *p* < 0.001), and all explanatory variables (with t-values = 6.4–12.8, and all *p’s* < 0.001,).

Importantly, the LMM analysis showed that when controlled for baseline well-being, groups differed significantly on T1 well-being, with a moderate effect-size (Table 2). Further, the LMM analyses showed that when controlled for baseline measures, the intervention group scored significantly lower on anxiety, depressive feelings (small effects), stress, and rumination (moderate effect-sizes), and significantly higher on gratitude as stable trait, positive reframing (small effects), and gratitude as mood (moderate effect).

**Table 2:** Estimated Marginal Means, between-group difference statistics of T1 and T2 controlled for T0, and T1-T2 within-group difference statistics

Scale	Range	Intervention group (IG)		Control Group (CG)		T1 between-group difference		Effect size		T2 between-group difference		T1-T2 within-group difference IG	
		T1 *M* (*SD*)	T2 *M* (*SD*)	T1 *M* (*SD*)	T2 *M* (*SD*)^a^	*F*	*p*	*d*	95% CI	*F*	*p*	*F*	*p*
Well-being	0–5	3.1 (0.7)	3.1 (0.8)	2.7 (0.8)	3.2 (0.8)	52.18	.000	.49	.36 to .63	1.51	.22	0.10	.76
Anxiety	0–21	5.9 (4.0)	5.5 (4.3)	6.9 (3.7)	5.2 (4.2)	16.34	.000	.28	.14 to .41	0.97	.33	1.05	.30
Depression	0–27	5.0 (4.1)	4.6 (4.4)	6.0 (3.7)	4.3 (4.2)	16.07	.000	.28	.14 to .41	0.75	.39	0.95	.33
Stress	0–40	15.0 (4.9)	14.6 (6.5)	17.3 (4.5)	14.8 (6.3)	48.69	.000	.48	.34 to .61	0.27	.60	0.48	.49
Explanatory variables													
Gratitude													
As trait	1–7	5.7 (0.7)	5.7 (0.8)	5.5 (0.7)	5.7 (0.8)	12.19	.001	.24	.10 to .37	0.26	.61	0.56	.46
Mood	1–7	5.6 (1.2)	5.7 (1.2)	5.2 (1.0)	5.9 (1.2)	30.57	.000	.38	.25 to .52	2.28	.13	1.57	.21
Positive reframing	4–16	12.6 (2.4)	12.7 (2.7)	12.0 (2.1)	12.7 (2.6)	15.71	.000	.27	.14 to .41	0.01	.92	0.41	.52
Rumination	0–60	25.0 (8.5)	23.5 (10.7)	28.4 (7.7)	23.7 (10.4)	37.01	.000	.42	.28 to .55	0.11	.75	2.18	.14

^a^Control group also completed the intervention at T2

At T2, when the waitlist control group had also completed the intervention, the control group improved to the level of the intervention group: no significant differences between groups remained on any of the outcome measures, and no within-group differences were found in the intervention group between T1 and T2.

### Moderation Analyses

The moderation test to explore whether the intervention was effective for people with various levels of distress, showed a non-significant interaction effect for both baseline anxiety and depression: *F*(2,717) = 0.52, *p* = 0.59, and *F*(2,717) = 0.30, *p* = 0.74, respectively. This indicates that the intervention effectiveness was not moderated by level of distress at the start of the intervention.

### Path Analyses

The contribution of gratitude and coping (i.e., positive reframing and rumination) on the efficacy of the intervention was analysed with simple pathway models (Table 3). For each model, path a, b, c were significant, the direct effect (c’) was smaller than the total effect (c) but remained significant, and the 95% CI of the indirect effects did not include 0. This indicates that the effect of the intervention was partially explained by gratitude, positive reframing and rumination.

**Table 3:** Unstandardized regression coefficients of simple path analyses of the effects of the intervention vs. waitlist control on well-being change scores (T1-T0), explained by gratitude, positive reframing and rumination change scores (T1-T0)

Explanatory variable	*a*	*b*	Total effect *c*	Direct effect *c’*	Indirect effect axb (95% CI)
Gratitude as trait	0.24***	0.27***	0.35***	0.29***	0.06 (0.03–0.09)
Gratitude as mood	0.57***	0.14***	0.35***	0.27***	0.08 (0.05–0.12)
Positive reframing	0.74***	0.08***	0.35***	0.29***	0.06 (0.03–0.09)
Rumination	−3.37***	−0.02***	0.35***	0.28***	0.07 (0.04–0.11)

### Dose–Response Relations

**Fig. 3:**
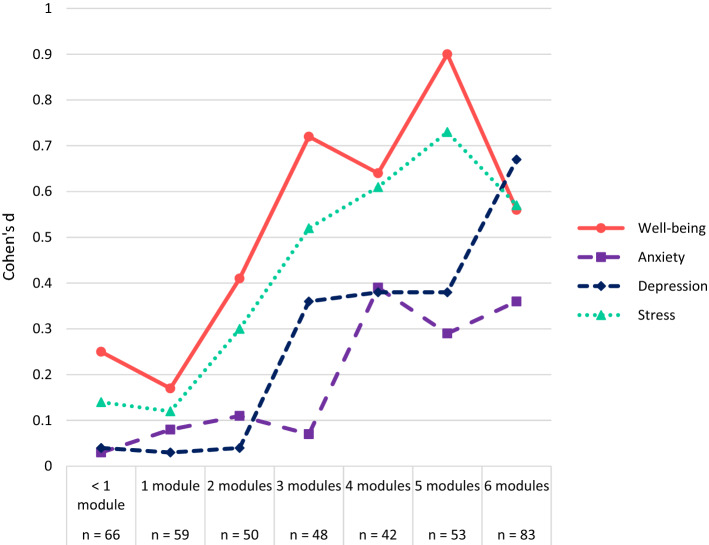
Cohen’s *d* of intervention group pre-post estimated marginal means for well-being and ill-being variables only

The number of modules finished was weakly related to change-scores in well-being (*r* = 0.21), anxiety (*r* = −0.17), depression (*r* = −0.27), and stress (*r* = −0.24, all *p*’s < 0.01). Figure 3 show trends in Cohens’ *d* of intervention group pre-post estimated marginal means. For well-being and stress, rather steady improvements of effect sizes are visible from 1–5 modules, with greatest improvements after finishing the first 2–3 modules. Anxiety improved greatest for people finishing module 4 (Grateful memories) but did not improve further after this. For depression, effect size improved greatly for users finishing module 3 (Expressing gratitude), and then again at module 6 (Grateful attitude).

### App Evaluations

Table 4 shows intervention group participants’ satisfaction with the app. Overall, users indicated being moderately satisfied with the app, and indicated the first module (Gratitude diary) and the second module (Take perspective) as most useful modules. Users were generally positive about language usage, and the amount of text, the number of modules, the design and the usability. The texts and exercises were thought to be appealing, but the daily quotes, videoclips, and notifications were only appealing for about half of the participants.

**Table 4:** Evaluations of the app: content, satisfaction and appeal

Client satisfaction	*Scale*	*M*	*SD*
	8–32	22.6	4.8

Module content	Most useful^a^		
1 Gratitude diary	61%		
2 Take perspective	53%		
3 Expressing gratitude	25%		
4 Grateful memories	23%		
5 Gratitude and misfortune	22%		
6 Gratitude attitude	25%		

Satisfaction	Bad/moderate	Reasonable	Good/very good
Language	4%	10%	86%
Amount of text	6%	17%	77%
Design	9%	18%	73%
Number of modules	11%	21%	69%
Usability	15%	21%	64%

Appealing	Not/somewhat	Largely/completely	
Texts	28%	72%	
Exercises	32%	68%	
Daily quotes	47%	53%	
Video’s	48%	52%	
Notifications	54%	46%	

^a^ There was no restriction on the number of modules to assign most useful

There were some technical issues during the study (e.g., issues with login, uploading photo, app crash, refreshing the app), and 26% of respondents felt this may have influenced their motivation to work with the app (not in Table). Most participants (71%) were satisfied with writing the exercises on paper, or on another device or in another app, but a minority (26%) would prefer an extra functionality to write in the app.

## Discussion

Previous findings showed the importance of gratitude as a psychological resource during the Covid-19 pandemic (Bono et al., 2020; Mead et al., 2021; Pellerin & Raufaste, 2020). The aim of the current study was to test the effectiveness of and satisfaction with a 6-week gratitude intervention in app format to improve mental health of people with reduced well-being in times of the Covid-19 pandemic. Furthermore, we investigated gratitude and coping as possible mechanisms of change, examined dose–response relationships, and evaluated the appeal of the intervention.

The online gratitude intervention was effective in improving mental health in the general population during the Covid-19 pandemic, and the effects remained after six weeks follow-up. Well-being improvements exceeded the effects previously found in meta-analyses of gratitude interventions (Davis et al., 2016; Dickens, 2017), which could be due to the longer duration and greater diversity of exercises in the current intervention (Bohlmeijer et al., 2020). At the same time, effects were smaller than previously found in the studies on online gratitude interventions using other formats (i.e., pdf-email format, or app combined with online training; Bohlmeijer et al., 2020; Heckendorf et al., 2019). This might be explained by the absence of additional guidance in the current intervention, besides email-based technical support. Guidance can improve effectiveness (Baumeister et al., 2014; Beatty & Binnion, 2016), but it simultaneously reduces the feasibility of scaling the intervention into population-wide implementation. Others have included alternative interactive aspects in their gratitude intervention that might be better scalable, such as connecting to other users to share gratitude experiences (Bono et al., 2020), or a single physical kick-off meeting (Van ’t Klooster et al., 2017). The impact of such alternatives on effectiveness remains to be investigated.

We found that intervention effects on well-being were not dependent on baseline anxiety or depression. This indicates that both people with mild and moderate distress benefit from the intervention and highlights the suitability of a gratitude intervention during difficult times. Furthermore, the intervention was also effective in reducing ill-being, although effects were smaller compared to well-being. This is in line with previous studies (Cregg & Cheavens, 2021; Jans-Beken et al., 2020), and with the idea that well-being and ill-being are related but separate concepts (Lamers et al., 2011a; Westerhof & Keyes, 2010). It is not surprising that people continued experiencing some degree of anxiety, depressive feelings, and stress during the continued pandemic and associated regulations. However, it has been suggested that because gratitude improves well-being, this could in turn have a buffering effect on anxiety and depression (Schotanus-Dijkstra et al., 2017; Waters et al., 2021), which may take longer to surface. Such temporality of effects should be further examined with longer follow-up measurements.

The effects of the intervention on well-being were partly explained by improvements in gratitude and effective coping (i.e., improved positive reframing and decreased rumination). These results are in line with previous work suggesting effective coping as a potential underlying mechanism to explain the effect of gratitude on mental health (Heckendorf et al., 2019; Lambert et al., 2012; Wood et al., 2007), which may be especially essential during the difficult circumstances such as a pandemic (e.g., Büssing et al., 2020; Jans-Beken, 2021). The findings suggest that gratitude, positive reframing, and rumination are processes of change in the effects of a gratitude intervention on mental health. However, it should be noted that our results could not establish temporal precedence (i.e., the assumption that changes in the explanatory variable occurs before changes in the outcome; Kendall et al., 2017), as we were limited to using only two timepoints. Furthermore, the pathways we found were only partial, leaving room for additional mechanisms at play. First, gratitude can be considered a positive emotion (McCullough et al., 2002), and a gratitude intervention may thus have a direct effect on mental health. Second, additional processes of change are for instance positive relationships, positive emotions, and hope (Algoe & Zhaoyang, 2016; Feng & Yin, 2021; Lambert et al., 2012), but these were not measured in the current study. Third, a recently developed model for sustainable mental health describes that barriers such as rumination and resources such as gratitude and positive reframing can improve sustained mental health through improving one’s ability to adapt (Bohlmeijer & Westerhof, 2021), suggesting a serial multiple mediation. Future research should shed light on these potential mechanisms and their relative explanatory power in the effect of gratitude interventions on mental health.

Besides showing gratitude and coping as mechanism of change, this study also revealed dose–response relationships. Finishing a greater number of modules was weakly related to better outcomes. This is in line with a recent meta-analyses, which showed that longer positive psychology interventions generally have larger effects (Carr et al., 2020), although this may not be extend to long-term effects (Koydemir et al., 2021). Further inspection of our results showed linear trends for well-being and stress, and a less gradual trend for ill-being, with steep improvements at certain modules. These results suggest that for the current intervention, an optimal dose may be between 4 and 6 modules, depending on the outcome. This is in line with a recent study showing gratitude intervention effects were larger at 4 weeks compared to at 2 weeks (Bohlmeijer et al., 2022), and this number of modules seems similar to a systematic review suggesting an optimal dose of 4–6 sessions for low intensity guided psychological self-help interventions (Robinson et al., 2020). However, the modules in the current intervention covered various gratitude exercises, making it impossible to disentangle the impact of simple repetition of exercises from effects of the diversity of the exercises, which may both contribute to effectiveness (Carr et al., 2020). Finally, while drop-out rates were comparable to previous studies (e.g., Heckendorf et al., 2019), only about a third fully adhered to the intervention goals. It is important to note that we may have been a bit too strict with our predefined intended app usage (i.e., 4 or more modules finished, spending at least 10 min per day for at least 5 days per week on the intervention). Indeed, there is a debate about what adherence measures to use, and some have suggested using number of finished modules as adherence (Gan et al., 2021), which would mean that more participants adhered. Still, a majority of participants did not reach module 4 or higher in the current study, which signals the need to further support adherence in order to unlock the full potential effectiveness of the intervention.

The evaluations of the users additionally gave some first suggestions for intervention improvements, for instance by reducing the number of notifications, and changing the video’s, and daily quotes. Other ways of improving adherence could be by incorporating additional persuasive elements, such as tailoring the notifications (Bidargaddi et al., 2018), or including personalization, praise, or a social role in the intervention by means of a supportive avatar (Kelders et al., 2012). That said, we found that overall, this gratitude app was satisfactory for the general population with reduced well-being, specifically concerning the content, used language, amount of text, number of modules, design and usability. The number of people applying to participate greatly exceeded our targeted sample size, and the satisfaction with the app was comparable to the pdf-email format gratitude intervention (Bohlmeijer et al., 2022), indicating the need for and suitability of such a positive psychology intervention in times of a pandemic with lockdowns and restrictions (Waters et al., 2021).

## Strengths and Limitations

This is the first study to test the effectiveness of a gratitude intervention in app format, during the Covid-19 pandemic. This study has several strengths, such as including a control, describing baseline differences, employing an intention-to-treat analyses, describing characteristics of participants lost to follow-up, and using a pre-specified analysis plan (Boggiss et al., 2020; Davis et al., 2016; Dickens 2017). Furthermore, we used an efficient study design, investigating both effectiveness and satisfaction, and the relatively big sample size (Dickens 2017) provided the opportunity for examining underlying mechanisms and dose–response relationships. However, there are also various limitations to this study.

First, we only included a waitlist control condition, not an active control condition, so we cannot make any inference on whether the gratitude app is more effective than other well-being apps. Second, we did not examine the added effect of an online app compared to other modes of delivery, nor did we test the effects of specific app features or persuasive elements. Third, the dose–response relations are based on drop-out, rather than assigned dosage, and should therefore be interpreted with caution. Especially in light of the low adherence rates, the results may be biased in the sense that the reported trends could be partially related to specific participant characteristics (e.g. having a stronger motivation for gratitude interventions) by the participants in the sample that adhered to the intervention. Further experiments are needed to unravel specific effects of delivery features and dose-effects (Koydemir et al., 2021). Fourth, this study only provides a limited indication of the longer-term effects of the intervention. The effects in the intervention group remained after 6 weeks, but in the absence of a true control group, we cannot assert the potential effects of some of the Covid-19 measures being slowly lifted during T2 assessments (National Institute for Public Health and the Environment, 2021). Still, the unambiguity of the results in both groups, and the absence of potential non-specific effects from guidance, support the effectiveness of the intervention.

Finally, there are some limitations concerning the sample. The sample consisted solely of Dutch and Belgian participants, and of mainly highly educated women, which reduces generalizability. The education level of respondents is not surprising given the recruitment channels and text-driven intervention, and female participation rates comparable to other studies (Bendtsen et al., 2020; Bohlmeijer & Westerhof, 2021; Schotanus-Dijkstra et al., 2017). Gender and education level were not related to intervention drop-out, but future studies should still examine how to make gratitude interventions appealing for more men and for people with practical education levels. It should also be noted that the sample included mainly people who experienced only limited detrimental effects of COVID-19, in terms of having (in)direct contact with an infection, loss of a loved one, and its self-reported impact on well-being. We additionally excluded people with severe anxiety and with (moderately) severe depressive symptoms, all of which making results only generalizable to people who were moderately impacted by the pandemic, and with minimal to moderate distress. On the other hand, experiencing low well-being was not an exclusion criterium, but instead stressed in the recruitment message. However, since the well-being of our sample was still below the Dutch norm group, we seem to have still included the targeted population.

The gratitude app that was developed for this study provides the first Dutch evidence-based (Bohlmeijer et al., 2020) and scientifically supported way to improve well-being through gratitude exercises, and is currently freely available in the Netherlands. Although some literature has suggested superior effectiveness of multi-component positive psychological interventions (Sin & Lyubomirsky, 2009), it has also been shown that people differ in their preferences for certain positive psychological exercises over others, which subsequently influences engagement (Schueller, 2010). Indeed, besides features of the activity, and features of the person, the specific person-activity fit has been reported to influence intervention effectiveness (Lyubormirsky & Layous, 2013; Proyer et al., 2015). Our gratitude app contributes to implementing a wide variety of evidence-based and tested positive psychological intervention apps, which offers people the opportunity to select exercises that fit their person to support their well-being. Future research should invest further in making such interventions also fit to groups who are currently largely overlooked, such as people with low SES (Faber et al., 2021), for example by using co-design strategies (Austin et al., 2022).

## Conclusion

The current study shows the potential of a six-week gratitude intervention app as an appealing and effective way to improve mental health of the general population in the context of an ongoing pandemic, through improving gratitude and effective coping, and with a higher dosage related to greater effectiveness. Our intervention was presented in a relatively simple Progressive Web App format without guidance, which can be easily scaled, to provide a cost-effective easily accessible means for supporting well-being (e.g., Bidargaddi et al., 2018; Mohr et al., 2010; Olff, 2015; Wei et al., 2020). This is especially important when restrictions (e.g., social distancing, lockdowns) and high demand prevent other forms of support to be readily available. The applicability of gratitude exercises during other times of collective distress (such as natural disasters), and during intense experiences of personal distress (such the grief of losing a loved-one, dealing with personal illness) remains to be investigated. But for now, the current study shows that practicing gratitude using a mobile application has potential to make a significant impact on the mental health of the general population, even during the difficult times of a pandemic.

## Data Availability

Data is available upon request.
